# Comparing static and outreach immunization strategies and associated factors in Uganda, Nov-Dec 2016

**DOI:** 10.11604/pamj.2019.32.123.16093

**Published:** 2019-03-15

**Authors:** Fred Nsubuga, Steven Ndugwa Kabwama, Immaculate Ampeire, Henry Luzze, Pande Gerald, Lilian Bulage, Opar Bernard Toliva

**Affiliations:** 1 Uganda National Expanded Program on Immunization, Ministry of Health, Kampala, Uganda; 2 Uganda Public Health Fellowship Program, Field Epidemiology Track, Ministry of Health, Kampala, Uganda

**Keywords:** Vaccination, vaccination coverage, survey, Uganda

## Abstract

**Introduction:**

the government of Uganda aims at reducing childhood morbidity through provision of immunization services. We compared the proportion of children 12-33 months reached using either static or outreach immunization strategies and factors affecting utilization of routine vaccination services in order to inform policy updates.

**Methods:**

we adopted the 2015 vaccination coverage cluster survey technique. The sample selection was based on a stratified three-stage sample design. Using the Fleiss formula, a sample of 50 enumeration areas was sufficient to generate immunization coverages at each region. A total of 200 enumeration areas were selected for the survey. Thirty households were selected per enumeration area. Epi-Info software was used to calculate weighted coverage estimates. facility

**Results:**

among the 2231 vaccinated children aged 12-23 months who participated in the survey, 68.1% received immunization services from a health unit and 10.6% from outreaches. The factors that affected utilization of routine vaccination services were; accessibility, where 78.2% resided within 5km from a health. 29.7% missed vaccination due to lack of vaccines at the health facility. Other reasons were lack of supplies at 39.2% and because the caretaker had other things to do, 26.4%. The survey showed 1.8% (40/2271) respondents had not vaccinated their children. Among these, 70% said they had not vaccinated their child because they were busy doing other things and 27.5% had not done so because of lack of motivation.

**Conclusion:**

almost 7 in 10 children aged 12-23 months access vaccination at health facilities. There is evidence of parental apathy as well as misconceptions about vaccination.

## Introduction

Globally, close to 1.5 million children die every year due to vaccine preventable diseases. Also an estimated 18.7 million infants are still not reached by routine immunization services with more than 60% of these living in 10 countries, Uganda inclusive [1]. Immunization however, is one of the most successful and cost-effective interventions at improving health outcomes around the globe [2-6]. High vaccine uptake rates specific to each vaccine preventable disease are needed by countries if community-level immunity is to be achieved and sustained [7]. In 2012, 194 World Health Organization (WHO) member states endorsed the Global Vaccine Action Plan (GVAP) and committed to ensure no one misses out on vital immunizations; with a target of 90% diphtheria-pertussis-tetanus third dose (DTP3) vaccination coverage in all countries by 2015 [8]. In 2013, the estimated global DTP3 coverage among children aged <12 months ranged from 75% in the WHO African Region to 96% in the Western Pacific and European regions [9]. The 2014 GVAP assessment report shows that progress is still slow. The government of Uganda through the Uganda Immunization Policy 2014, National Development Plan and National Health Policy aims at reducing childhood morbidity and mortality through provision of immunization services. It was declared to have eliminated indigenous wild polio in 2006 and maternal and neo-natal tetanus in 2011. Available administrative data for 2014 indicate a DPT3 coverage of 101%, with a DPT1-DPT3 drop-out rate of 7.2% which is within acceptable range, measles at 96% and BCG (90%). Routine immunization (RI) services are delivered through fixed-post sites (i.e, within the health facility) and enhanced by outreach services for populations living in areas with limited access to fixed services [10, 11]. Outreaches are planned, regular and periodic single-day visits by qualified staff from a health facility to populations located 5-15 km from the facility. Apart from provision of immunization services, outreaches present opportunities to provide women, children and their families with other vital interventions, such as vitamin A supplementation, deworming tablets, and insecticide-treated nets [12]. Although Uganda has done well to achieve this high coverage, the contribution of each of these strategies (fixed-post and outreach services) to the immunization coverage per year is not documented. The Ministry of Health (MOH) and partners have invested huge sums of resources in routine immunization specifically to support districts in conducting outreaches in the community. However, the national reporting system captures aggregated data for routine immunization and is therefore unable to differentiate the contribution of each of these strategies to the general immunization performance. This study intended to estimate the proportion of children reached through static and outreach immunization strategies and to describe the factors that affect utilization of routine immunization services in order to generate evidence for planning and policy updates.

## Methods

**Figure 1:**
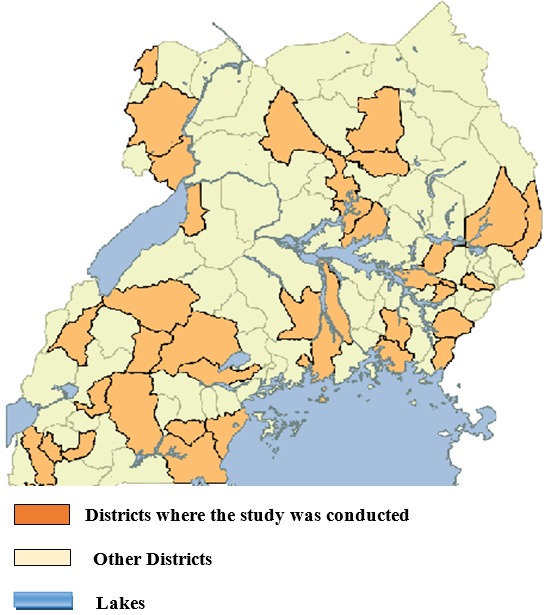
districts where the study was conducted

**Study area:** the study was conducted in the 4 regions of Uganda comprised of: northern, western, eastern and central regions. Each region is divided into several districts, Figure 1. These districts are divided into municipalities, town councils, sub-counties, parishes and villages/enumeration areas. Villages consist of several households. During this study, each enumeration area constituted a cluster.

**Study design:** this study used the 2015 WHO vaccination coverage cluster survey method [13]. Data was collected between November and December 2016. The target population were all children aged 12-23 months. The survey used the 2014 population and housing census frame managed by the national statistics office of Uganda.

### Sampling and sample size determination

**Sample size and sampling procedure:** the sample size estimate was computed to provide nationwide estimates for routine vaccination coverage. The estimates were reported for the 4 regions of the country according to the 2014 census. We computed the sample size using the Fleiss formula [14]. A sample of 1500 households was sufficient to generate immunization levels at each of the 4 regions with a national sample of 6000 households. Fifty enumeration areas (EAs), each with a sample of 30 households were selected for each of the 4 regions, giving a total of 200 EAs for the survey.

**Stratification:** in order to increase the efficiency of the sample design, the sampling frame was divided into 15 strata which are as homogeneous as possible.

**Sample selection procedure for districts, enumeration areas (EAs) and households:** the sample selection procedure was based on a stratified three-stage sample design. At the first sampling stage, a sample of districts was selected systematically with probability proportionate to size (PPS) within each stratum. Prior to the second sampling stage, the sampling frame of EAs within each stratum was geographically ordered to provide implicit stratification and obtain a sample that is geographically representative within each sampling stratum. During the second stage, a sample of EA was selected with PPS within each district and within each selected EA a sample of 30 households was selected.

**Assessment of vaccination status and strategy of delivery:** we assessed vaccination status and strategy of delivery for each of the children aged 12-23m in the sampled household. Evidence of vaccination was based on visual inspection of an immunization card or book, or from recall /history. Caretakers were asked whether their children had received vaccination at health unit or outreach session. Static immunization services were described as routine immunization services conducted daily at health facilities/hospitals. Outreach immunization services were described as planned and regular visits by qualified staff from a health unit to populations located more than 5km from the facility to provide vaccinations. Evidence for immunization was based on visual inspection of an immunization card or book, or from recall /history. Coverage was calculated on all routine doses received by the time of the study.

**Data collection:** data was collected using a structured questionnaire, where the interviewer was responsible for contacting the respondent, for asking questions and for recording the responses. The regional supervisors were responsible for checking the completed forms at the end of the day to ensure that they are properly filled.

**Statistical analysis:** data was collected using electronic data collection tablets using Kobo Collect software. These were then downloaded in Microsoft Excel format and exported to Epi Info. We used Epi-Info software to calculate weighted coverage estimates and other descriptive statistics.

**Ethics consideration:** ethical review was sought from Makerere School of Public Health Institutional Review Board (IRB) under protocol 431. The survey was also registered with the Uganda National Council for Science and Technology and approved under number (SS 4128). Administrative clearance was sought from the respective districts. Each study participant was informed about the purpose of the study, they were made to understand that participation in the study was on a voluntary basis and a participant was free to withdraw at any time during the study. They were also told that they are free to refuse to answer any of the questions without any consequences. Informed consent was sought from respondents or, assent for under-aged respondents in a language which they understand.

## Results

From the targeted 6000 households nationally, 5563 households were accessed which yields a response rate of 92.7%. The majority of the parents/caretakers were mostly female 85.9% (4795/5563) and more than half 53.3% (2977/5563) had primary level education (Table 1). The weighted total number of eligible children for the four regions was 1,483,841. This included 471969.6 in the central region, 237656 in the eastern region, 185091.6 in the northern and 589124.1 in the western region. For central and eastern regions, the immunization coverage was less than 90% for 6 of the 11 antigens. In the northern and western regions, the immunization coverage was less than 90% for only two of the antigens (Table 2). Among the persons who participated in the survey, 68.1% (1520/2231) received immunization services from a health unit alone, 10.6% (237/2231) received the immunization services from outreaches alone and 21.3 (474/2231) received immunization services from a health unit and or outreach on any of the occasions. In addition, we found that 71.5% (1624/2271) were fully immunized, 21.7% (607/2271) were partially immunized and 1.8% (40/2271) were not vaccinated. Immunization services were mostly received from the health unit for all antigens (Table 3). We also assessed factors that affect utilization of routine immunization services and found that 78.2% (1761/2252) of the respondents resided in an area located within 5km of a health facility. Most of the respondents accessed the health facility by walking 70.4% (1600/2252). Almost a third 29.7% (670/2252) said that they had missed vaccination services due to lack of vaccines at the health facility (Table 4). In this survey, 23.5% (531/2259) of the respondents said their child had never missed vaccination services. Among these, the most common reason care takers gave for missing immunization services was the lack of supplies such as injections, or fuel; 39.2% (208/531). Other common reasons were because the child was sick; 24.5% (130/531) and because the caretaker had other things to do; 26.4% (140/531).

**Table 1:** characteristics of participants during a survey conducted in the 4 regions of Uganda, Nov-Dec 2016

Characteristic	n	%
**Sex of parent/ caretaker**		
Female	4795	85.9
Male	787	14.1
**Region of respondents**		
Central	1371	24.6
Eastern	1338	24.0
Northern	1370	24.5
Western	1503	26.9
**Number of households per region**		
Central	1366	24.6
Eastern	1335	24.0
Northern	1363	24.5
Western	1499	26.9
**Number of children below 5 years of age**		
0	24	0.4
1	2358	42.4
2	2249	40.4
3	751	13.5
> 3	181	3.3
**Number of children in Household between 12-23 months**		
0	3338	59.7
1	2135	38.2
2	112	2.0
3	4	0.1
**Level of education completed by caretaker**		
No education	1209	21.7
Primary school	2977	53.3
Secondary education	1141	20.4
Tertiary education	256	4.6
**Sex of children between 12-23 months of age**		
Female	1219	53.7
Male	1052	46.3
**Child has received immunization before**		
Yes	2231	98.2
No	40	1.8
**Child has an immunization card**		
Yes	2042	91.5
No	189	8.5

**Table 2:** weighted immunization coverages by antigen, during a survey conducted in Uganda, Nov-Dec 2016

	Central	Eastern	Northern	Western
Antigen	n (%)	n (%)	n (%)	n (%)
**BCG**	**453,151 (96)**	**225,495 (95)**	**184,730 (99.8)**	**556,297 (94))**
**OPV1**	**437,205 (93)**	220,939 (93)	182,994 (99)	567,977 (96)
OPV2	425,081 (90)	218,898 (92)	177,571 (96)	565,469 (96)
OPV3	39,726 (84)	213,007 (90)	170,770 (92)	551,847 (94)
DPT1	432,992 (92)	221,218 (93)	183,527 (99)	560,337 (95)
DPT2	422,676 (90)	219,664 (92)	178,106 (96)	551,782 (94)
DPT3	396,620 (84)	209,543 (88)	169,692 (92)	539,796 (92)
PCV1	426,130 (90)	213,162 (90)	180,580 (98)	544,246 (92)
PCV2	418,834 (89)	204,240 (86)	173,910 (94)	535,276 (91)
PCV3	392,777 (83)	197,216 (83)	164,973 (89)	522,118 (89)
Measles	339,610 (72)	176,694 (74)	156,218 (84)	480,470 (82)

**Table 3:** source of immunization services by antigen, during a survey conducted in Uganda, Nov-Dec 2016

Antigen	Health Unit n (%)	Outreach n (%)	Both Health unit and outreach n (%)	Total
BCG	1,711 (78.8)	203 (9.4)	256 (11.8)	2,170
OPV1	1,628 (74.7)	279 (12.8)	271 (12.4)	2,178
OPV2	1,523 (71.3)	331 (15.5)	282 (13.2)	2,136
OPV3	1,443 (69.6)	328 (15.8)	301 (14.5)	2,072
DPT1	1,614 (74.7)	280 (13.0)	266 (12.3)	2,160
DPT2	1,550 (73.2)	310 (14.6)	258 (12.2)	2,118
DPT3	1,490 (72.6)	305 (14.9)	256 (12.5)	2,051
PCV1	1,590 (74.9)	277 (13.0)	256 (12.1)	2,123
PCV2	1,514 (73.1)	302 (14.6)	256 (12.4)	2,071
PCV3	1,456 (72.8)	290 (14.5)	250 (12.5)	2,000
Measles	1,328 (70.5)	311 (16.5)	245 (13.0)	1,884

**Table 4:** factors that affect utilization of routine immunization services, during a survey conducted in Uganda, Nov-Dec 2016

Distance (km) to the nearest health facility	n	%
<5	1,761	78.2
5 - 10	455	20.2
>10	36	1.6
**Ways of accessing the nearest health facility**		
Walking	1,600	70.4
Using motorcycle	356	15.7
I use a bicycle	231	10.2
Using a taxi	68	3.0
I use my own car	15	0.7
I use a boat	1	0.04
**Health workers communicate well during immunization sessions**		
Yes	2,134	94.5
No	125	5.5
**Child has missed vaccination due to lack of vaccines at health facility**		
Yes	670	29.7
No	1,589	70.3
**Child has ever missed vaccination**		
Yes	531	23.5
No	1,728	76.5

## Discussion

Among the children that participated in the survey, we found that 71.5% were fully vaccinated, 26.7% were partially vaccinated while 1.8% had not received any form of vaccination. This finding is higher in comparison with previous National Household surveys that reported lower percentage of children fully vaccinated at the time of the survey. The Uganda Demographic Health survey (UDHS) 2011 [15] reported that 52% of children aged 12-23 months were fully vaccinated at the time of the survey while the UDHS 2006 [16] reported that 46% of children were fully vaccinated at the time of the survey. The immunization coverage by antigen was also higher for all antigens in comparison with findings from the UDHS 2011 [15]. The increase in the percentage of children accessing immunization services over the past 15 years could be attributed to an improvement in the health system infrastructure where more health centers have been built at lower levels of the health system. This also goes hand in hand with the improvement in the human resources for health. In addition, there has been an increase in the funding of EPI activities such that vaccines have increased in quantity but also the efficiency with which they are availed to the population. In fact, our results show that an average of about 12% of the children in this survey received immunization services through outreach. Furthermore, over the past 10years, there has been an increase in the frequency of supplementary immunization activities during the year which also enhance routine immunization services. The immunization coverage was higher than 90% for BCG, OPV1 and OPV2 for all the regions. For PCV3 and Measles however, the immunization coverage was lower than 90% for all the regions. BCG is received at birth, OPV1 at 6 weeks and OPV2 at 10 weeks. PCV3 is received at 14 weeks, and Measles at 9 months. This difference in coverage between the antigens received earlier on in life compared to those received later might be attributed to the parental apathy that comes with having to go to the health center several times to receive the vaccines. Parental apathy has been shown to be a barrier to timely childhood immunization especially in poor settings [17]. The perception of parental attitudes by health care providers has also been shown to be important in immunization service delivery [18]. As health care workers dispense vaccinations in the early stages of life, they should emphasize to care takers and parents the importance of completing the immunization schedule by the age of one year. This finding also underscores the importance of strengthening routine vaccination activities during national immunization days. Immunization services were mostly received from the health unit for all antigens. This was followed by outreach only then both health unit and outreach. Investment in the improvement of the health care system infrastructure particularly the health care facilities might prove to be very useful in improving the immunization coverage. In terms of cost however, a study done in Bangladesh found that the cost of providing immunization services through outreach was significantly lower than the health facility [19]. Considering that only 10.6% of children received vaccinations through outreach immunization services, it might prove cost-effective in the long run to fortify the immunization services at health facilities with outreach immunization activities. Also, regular periodic outreaches by health care workers have been shown to significantly increase immunization service utilization [20].

It is interesting to note however that BCG was the only antigen for which the percentage of children who attended both the health unit and or outreach for vaccination services was higher than those that received it through outreach only. According to the UDHS 2011 [15], 57% of births in Uganda take place in a health facility. BCG is given to new born children at birth and this might be why most children access it at the health facility. This might explain the higher access of this particular antigen at the health facility compared to outreach. Delivery at health centers provides a unique opportunity through which the BCG vaccine can be given to children. However, there still remains a significant percentage of children born away from a health facility. Village health team members and other community health workers need to engage community members to identify newly born children to avail them with vaccines they might not have had the opportunity to receive because they were delivered away from the health facility. We also assessed the factors associated with the utilization of routine immunization services and found that 78.2% of the respondents resided in an area located within 5km of a health facility. According to the Uganda National Housing Census (UNHS) 2009/2010 [21], the goal of the Health Sector Strategic Plan was to increase people's accessibility to health services such that every individual is within a 5km walking distance to a health facility. This means that the majority of persons in our survey had access to a health facility. Almost a third 29.7% said that they had missed vaccination services due to lack of vaccines at the health facility. This finding is corroborated by the situation analysis of the UNEPI comprehensive multi-year plan [22] which noted that inadequate logistics, poor cold chain maintenance and inadequate capacity for the management and delivery of the immunization services were major barriers to the achievement of high immunization coverage in Uganda. Funding for immunization activities should take into account the logistical challenges related to the cold chain so that vaccines are received at health facilities promptly and in the right quality and quantity. Among the participants in the survey, 26.4% mentioned that they were busy with other things and of those who had not vaccinated their children, 20% said that it was because the vaccination was not safe and 27.5% said it was because of lack of interest or motivation. Our findings are similar to those of Babirye *et al.* [23] who carried out qualitative investigations and found that the barriers to utilization of immunization services in Uganda were inconvenient schedules, time constraints and a fear of negative side effects of vaccination. A failure to appreciate the benefits of immunization was also one of the reasons immunization coverage in Uganda dropped drastically between the year 1996 and 2000 [22]. Sensitization and awareness need to be done both at health facilities and community levels to dispel any misconceptions and misunderstandings about the benefits of vaccination. The strength of this study lies in the big sample size. Limitation; we did not conduct analytical statistics and hence the factors presented in our study do not prove causality.

## Conclusion

Almost 7 in 10 children aged 12-23 months access immunization at health facilities. There is evidence of parental apathy as well as misconceptions and misunderstandings about the objective of vaccination. Sensitization and awareness need to be strengthened both at health facilities and community levels to dispel any misconceptions and misunderstandings about the benefits of vaccination. The government needs to invest in improving the health care system infrastructure particularly health facilities.

### What is known about this topic

- Routine immunization is the sustainable, reliable and timely interaction between the vaccine, those who deliver it and those who receive it to ensure that every child is fully immunized against vaccine-preventable diseases before the first birth day;
- Routine immunization services are delivered through fixed-post sites to populations within 5km and by outreach services for populations living in areas more than 5km;
- Political, social-economic, cultural and health systems factors may deter parents from demanding or accessing vaccination services.

### What this study adds

- This study showed that more children are vaccinated at health facilities compared to outreaches;
- It gives us an estimate of the regional vaccination coverages by antigen.

## Competing interests

The authors declare no competing interests.
